# Dynamic analysis of slant cracked rotor system considering nonlinear oil film force

**DOI:** 10.1371/journal.pone.0294293

**Published:** 2024-01-25

**Authors:** Qiang Ding, Zhiguo Feng, Yu Zhang, Weipeng Sun

**Affiliations:** 1 Chn Energy Dadu River Repair&Installation Co., Ltd, Leshan, Sichuan, China; 2 State Key Laboratory of Eco-Hydraulics in Northwest Arid Region, Xi’an University of Technology, Xi’an, Shannxi, China; Institute of Space Technology, PAKISTAN

## Abstract

In this paper, considering the combined effects of nonlinear oil film forces and cracks on the rotor-bearing system, the differential equations of motion with 4 degrees of freedom are established by Lagrangian method. Then, the Lundgren-Kutta method is used to solve them and the results of the model are compared with the experimental data. The study demonstrate that the cracked rotor-bearing system is relatively stable at subcritical speeds, mostly in the period-1 motion. But near 1/3 of the critical speed, there is an inner loop in its whirl orbit and a significant increase in the 2x frequency component. When the system speed rises to the region near 1/2 of the critical speed, though the bifurcation motion and a relatively high 2x frequency can be observed, there are no other reliable fault characteristics. The study proves that the rotor crack fault diagnosis method based on the whirl orbits is convincing for slant cracked rotors.

## Introduction

Rotating machinery is frequently subject to various failures owing to material and processing defects, unfavorable condition and faulty operation. However, even for optimally designed rotor systems, the excessive long-term loading will lead to slant cracking [1] for the increasing local stresses. The existence of these cracks will cause an asymmetry in the rotor stiffness and an increase in 2× and higher harmonics [2, 3], which eventually leads to abnormal vibration in the system. More seriously, the abnormal vibration can further expand these cracks and induce a negative chain reaction. Therefore, early detection and diagnosis of rotor cracks is significant to avoid accidents and minimize losses.

Theoretical studies on crack opening/closing (breathing) have been carried out early, which are represented by Gasch’s hinge model [4] and Mayes’s cosine model [5]. These theoretical models not only well describe the variation of the crack stiffness with the rotational period, also provide a basis for the study of low-frequency vibration characteristics for the slant cracked rotor and the whirl orbit under 2×harmonic [6].

There have been many researches on rotors with slant crack. Iwamonji proposed that the crack breathes with the torsional vibration of the shaft [7], and further employed the cosine function cos(*ω*_T_) to characterize the rotor stiffness variation. Prabhakar et al. [8] investigated the vibration characteristics of the rotor with slant crack passing through its bending critical speed, and by finite element method, they pointed out that when the slant crack exists, the subharmonic and superharmonic frequency components corresponding to torsional vibration frequency are centered on the critical speed of the rotor system. In recent years, the popularity of fracture mechanics has further triggered scholars to study the time-varying stiffness of cracked rotors [9, 10]. However, these studies are not a reliable basis for crack diagnosis because rotor stiffness and intrinsic frequency are less affected by cracks. While the rotor’s dynamic response is more sensitive to the change in crack depth and stiffness [10]. The direction of cracks (transverse and slant cracks, etc.) can also be distinguished by rotor’s vibration characteristics under torsional vibration excitation [11, 12]. Additionally, there are several studies of whirl orbits for cracked rotors in the subcritical speed region, which provide a more intuitive approach to crack diagnosis [13–15]. However, research or experimental validation of the whirl orbit of slant cracked rotors is few. This paper will complement the transverse response and the whirl orbit of slant cracked rotor.

In practical engineering applications, large rotating machinery is mostly equipped with sliding bearings, and the effect of nonlinear oil film force on the stability of the rotor-bearing system is difficult to predict. As early as 1976, Botman [16] observed the non-synchronous vibration in a rigid rotor-damper system when the speed exceeded 2 times the critical speed of the rotor. Chang-Jian et al. [17] studied a flexible rotor system supported by a short bearing, and the results proved that the bearing parameters have an important influence on the movement of the rotor-bearing system. Zhang et al. [18] established a flexible rotor test platform, and found that reducing the bearing ellipticity can attenuate or even eliminate resonance and self-excited vibration when the speed is close to two times the critical speed. Moreover, the effect of nonlinear oil film forces on the rotor bearing system can’t be ignored. Wan et al. [19] demonstrated that nonlinear oil film forces have a greater effect on the system, by comparing the effects of oil film forces, cracks and friction in the rotor bearing system. Based on the flexible cracked rotor-bearing system model,Ferjaoui et al. [20] proposed that the presence of rotor cracks affects the spinning orbit of the sliding bearings, and further reduces the motion stability, They also noted that the presence and increase of 1/2 and 1/3 frequency components can prove the presence of cracks.

Therefore, to investigate the effect of nonlinear oil film force on the slant cracked rotor system, a mathematical model of the cracked rotor-bearing system considering nonlinear oil film force was established, the stiffness variation was described by the cosine model, and the model was solved by the Runge-Kutta method. A rotor bearing test rig was developed and relevant experiments were conducted, the test results verified the accuracy of model. The research mainly focuses on the characteristics of rotor-bearing frequency and rotating orbit.

## Mathematical modeling

### Rotor bearing system

The study was exempt from institutional ethics committee approval.

**Fig 1:**
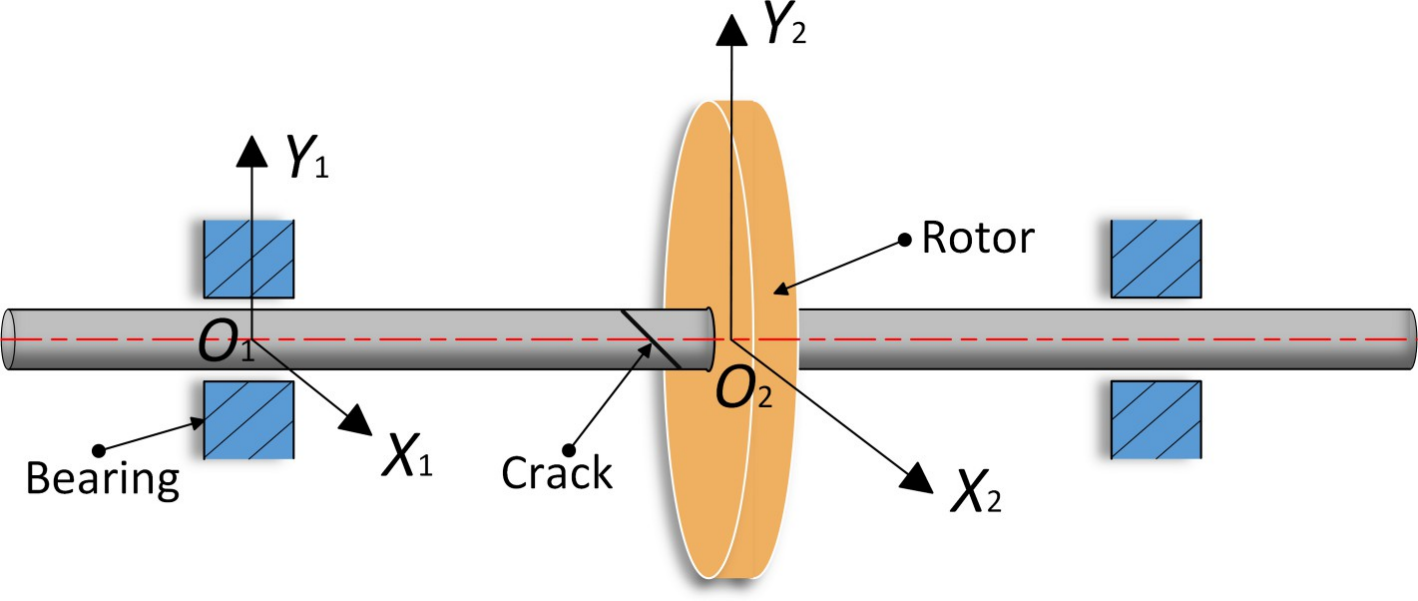


Fig 1 shows a cracked rotor-bearing system, which consists of a rotor, a shaft with a diagonal crack, and a pair of sliding bearings supporting the shaft. The sliding bearings are located at each end of the shaft, the diagonal crack and the rotor are located in the middle of shaft. To facilitate the theoretical analysis of system, the model introduces a two-coordinate system *X*_1_*O*_1_*Y*_1_ and *X*_2_*O*_2_*Y*_2_, where *O*_1_ and *O*_2_ are the shape centers of the rotor and the plain bearings, respectively.

The force analysis shows that, the rotor-bearing system is subjected to nonlinear oil film force [21], gravity, and centrifugal force caused by eccentricity. Compared with the horizontal and vertical deformations caused by oil film force and mass eccentricity, the shear deformations caused by torsional force and gyroscopic coupling are relatively small. To simplify the simulation, the Lagrange method is applied to establish the differential equations of the dynamics for rotor-bearing system in the two-coordinate systems *X*_1_*O*_1_*Y*_1_ and *X*_2_*O*_2_*Y*_2_, which are shown as follows:

$$\begin{array}{c} \left\{ \begin{array}{c} m_{1}{\ddot{x}}_{1}+c_{1}{\dot{x}}_{1}+k_{0}\left(x_{1}-x_{2}\right)/2=F_{x}\\ m_{1}{\ddot{y}}_{1}+c_{1}{\dot{y}}_{1}+k_{0}\left(y_{1}-y_{2}\right)/2=F_{y}-m_{1}g\\ m_{2}{\ddot{x}}_{2}+c_{2}{\dot{x}}_{2}+k_{xx}\left(x_{2}-x_{1}\right)+k_{xy}\left(y_{2}-y_{1}\right)=m_{2}e\omega^{2}\;\text{cos}\left(\omega t+\varphi \right)\\ m_{2}{\ddot{y}}_{2}+c_{2}{\dot{y}}_{2}+k_{yy}\left(y_{2}-y_{1}\right)+k_{xy}\left(x_{2}-x_{1}\right)=m_{2}e\omega^{2}\;\text{sin}\left(\omega t+\varphi \right)-m_{2}g \end{array}\right. \end{array}$$ (1)

where *m*_1_ and *m*_2_ are the masses of the bearing and disc, respectively, *c*_1_ and *c*_2_ are the damping coefficients of the bearing and disc, *ω* a is the system rotation speed, *φ* is the angle between the gravitational eccentricity and the crack direction, *g* is the acceleration of gravity, *F*_*x*_ and *F*_*y*_ are the nonlinear oil film forces in the *X* and *Y* directions, *k*_0_ is the stiffness of uncracked shaft, *e* is the eccentricity of the mass of the rotor, *k*_*xx*_, *k*_*yy*_, *k*_*xy*_ represent the stiffness and coupling stiffness of the cracked rotor in the fixed coordinate system.

### Nonlinear oil film force

To calculate the complex oil film forces, the classical theory of Capon’s circumferential bearings is employed to establish the Reynolds equation for short bearings,denoted as [22],

$$\begin{array}{c} \begin{array}{c} \frac{\partial }{\partial S}(\frac{H^{3}}{\mu }\frac{\partial P}{\partial S})+\frac{\partial }{\partial Z}(\frac{H^{3}}{\mu }\frac{\partial P}{\partial Z})=6U_{0}\frac{\partial H}{\partial S}+12\frac{\partial H}{\partial t} \end{array} \end{array}$$ (2)

where *H*, *P*, *μ* and *R* denote the oil film thickness, oil film dynamic pressure, dynamic viscosity and rotational radius, respectively. And *U*_0_ and *S* represent the tangential and circumferential velocities of the rotating axis, respectively. The dimensionless approach is introduced to generalize the Reynolds equation as

$$\begin{array}{c} \begin{array}{c} \frac{\partial }{\partial \theta }(h^{3}\frac{\partial p}{\partial \theta })+(\frac{R}{L}{)}^{2}\frac{\partial }{\partial z}(h^{3}\frac{\partial p}{\partial z})=\frac{\partial h}{\partial \theta }+2\frac{\partial h}{\partial \tau } \end{array} \end{array}$$ (3)

where *L* is the bearing length, $z=\frac{Z}{L}(-\frac{1}{2}\leq z\leq \frac{1}{2})$, $s=\frac{S}{L}$, $p=\frac{P}{6\mu \omega (R/C)}$ and $h=\frac{H}{C}$. The approximate relationship between the oil film thickness and the radius of the rotating axis can be expressed as [21]

$$\begin{array}{c} h=1-x\;\text{cos}\;\theta -y\;\text{sin}\;\theta \end{array}$$ (4)

Since the bearing length L is much smaller than the diameter. The variation of the oil film pressure in the circumferential direction is quite small compared to the longitudinal direction. Combining Eqs (3) and (4), the Reynolds equation can be simplified as

$$\begin{array}{c} \begin{array}{c} (\frac{R}{L}{)}^{2}\frac{\partial }{\partial z}(h^{3}\frac{\partial p}{\partial z})=x\;\text{sin}\;\theta -y\;\text{cos}\;\theta -2\left(x^{\prime }\;\text{cos}\;\theta +y^{\prime }\;\text{sin}\;\theta \right) \end{array} \end{array}$$ (5)

The non-dimensional dynamic oil film pressure can be further calculated as

$$\begin{array}{c} p=\frac{1}{8}(\frac{L}{R}{)}^{2}\frac{x\;\text{sin}\;\theta -y\;\text{cos}\;\theta -2(x^{\prime }\;\text{cos}\;\theta +y^{\prime }\;\text{sin}\;\theta )}{{\left(1-x\;\text{cos}\;\theta -y\;\text{sin}\;\theta \right)}^{3}}\left(4z^{2}-1\right) \end{array}$$ (6)

Furthermore, according to Eq (6), the initial angle *α* of the positive oil film pressure can be computed as

$$\begin{array}{c} \alpha ={\text{tan}}^{-1}\frac{y+2x^{\prime }}{x-2y^{\prime }}-\frac{\pi }{2}\text{sin}\frac{y+2x^{\prime }}{x-2y^{\prime }}-\frac{\pi }{2}\text{sign}\left(y+2x^{\prime }\right) \end{array}$$ (7)

Therefore, the total oil-film forces can be obtained as

$$\begin{array}{c} F_{x}=-[6\mu \omega (\frac{R}{C}{)}^{2}RL]\int_{\alpha }^{\alpha +\pi }\int_{-1/2}^{1/2}p\;\text{cos}\;\theta dzd\theta \\ F_{y}=-[6\mu \omega (\frac{R}{C}{)}^{2}RL]\int_{\alpha }^{\alpha +\pi }\int_{-1/2}^{1/2}p\;\text{sin}\;\theta dzd\theta \end{array}$$ (8)

Substituting Eq (6) into Eq (8), the equations for *f*_*x*_ and *f*_*y*_ are obtained as follows

$$\begin{array}{c} \begin{array}{c} f_{x}=\frac{1}{\sigma }F_{x}=2\int_{\alpha }^{\alpha +\pi }\frac{\left(x-2y^{\prime }\right)\text{sin}\;\theta \;\text{cos}\;\theta -\left(y+2x^{\prime }\right){\text{cos}}^{2}\;\theta }{{\left(1-x\;\text{cos}\;\theta -y\;\text{sin}\;\theta \right)}^{3}}d\theta \\ f_{y}=\frac{1}{\sigma }F_{y}=2\int_{\alpha }^{\alpha +\pi }\frac{\left(x-2y^{\prime }\right){\text{sin}}^{2}\;\theta -\left(y+2x^{\prime }\right)\text{cos}\;\theta \;\text{sin}\;\theta }{{\left(1-x\;\text{cos}\;\theta -y\;\text{sin}\;\theta \right)}^{3}}d\theta \end{array} \end{array}$$ (9)

where the Sommerfeld number *σ* is given by $\sigma =\mu \omega RL(\frac{R}{C}{)}^{2}(\frac{L}{2R}{)}^{2}$. To compute the integral, the non-dimensional oil film force is again rewritten as

$$\begin{array}{c} \begin{array}{cc} f_{x} & =2\left(x-2y^{\prime }\right)I_{3}-2\left(y+2x^{\prime }\right)I_{1}\\ f_{y} & =2\left(x-2y^{\prime }\right)I_{2}-2\left(y+2x^{\prime }\right)I_{2} \end{array} \end{array}$$ (10)

where *I*_1_, *I*_2_ and *I*_3_ are expressed as

$$\begin{array}{c} I_{1}\left(x,y,\alpha \right)\begin{array}{c} =\int_{\alpha }^{\alpha +\pi }\frac{{\text{cos}}^{2}\;\theta }{{\left(1-x\;\text{cos}\;\theta -y\;\text{sin}\;\theta \right)}^{3}}d\theta \end{array}\\ I_{2}\left(x,y,\alpha \right)=\int_{\alpha }^{\alpha +\pi }\frac{{\text{sin}}^{2}\;\theta }{{\left(1-x\;\text{cos}\;\theta -y\;\text{sin}\;\theta \right)}^{3}}d\theta \\ I_{3}\left(x,y,\alpha \right)=\int_{\alpha }^{\alpha +\pi }\frac{\text{sin}\;\theta \;\text{cos}\;\theta }{{\left(1-x\;\text{cos}\;\theta -y\;\text{sin}\;\theta \right)}^{3}}d\theta \end{array}$$ (11)

Based on the Leibniz integral rule [23], *I*_1_, *I*_2_ and *I*_3_ are calculated as

$$\begin{array}{c} \begin{array}{c} I_{1}=\frac{1}{2}\frac{\partial^{2}G}{\partial x^{2}}+\frac{\partial g(\alpha )}{\partial x}\frac{\partial \alpha }{\partial x}-\frac{\partial g(\alpha +\pi )}{\partial x}\frac{\partial \alpha }{\partial x}+\frac{1}{2}g\left(\alpha \right)\frac{\partial^{2}\alpha }{\partial x^{2}}+\frac{1}{2}g\left(\alpha +\pi \right)\frac{\partial^{2}\alpha }{\partial x^{2}}\\ I_{2}=\frac{1}{2}\frac{\partial^{2}G}{\partial y^{2}}+\frac{\partial g(\alpha )}{\partial y}\frac{\partial \alpha }{\partial y}-\frac{\partial g(\alpha +\pi )}{\partial y}\frac{\partial \alpha }{\partial y}+\frac{1}{2}g\left(\alpha \right)\frac{\partial^{2}\alpha }{\partial y^{2}}+\frac{1}{2}g\left(\alpha +\pi \right)\frac{\partial^{2}\alpha }{\partial y^{2}}\\ I_{3}=-\frac{\partial g(\alpha +\pi )}{\partial x}\frac{\partial \alpha }{\partial y}+\frac{1}{2}[\frac{\partial^{2}G}{\partial x\partial y}+\frac{\partial g(\alpha )}{\partial y}\frac{\partial \alpha }{\partial x}-\frac{\partial g(\alpha )}{\partial x}\frac{\partial \alpha }{\partial y}+g\left(\alpha \right)\frac{\partial^{2}\alpha }{\partial x\partial y}-g\left(\alpha +\pi \right)\frac{\partial^{2}\alpha }{\partial x\partial y]} \end{array} \end{array}$$ (12)

In the plane coordinate system *X*_1_*O*_1_*Y*_1_, substituting Eq (12) into Eq (10), the nonlinear oil film forces *F*_*x*_, *F*_*y*_ applied to the rotating axis are given by

$$\begin{array}{c} \left\{ \begin{array}{c} F_{x}=\sigma f_{x}=-\sigma \frac{\sqrt{{\left(x_{1}-2{y^{\prime }}_{1}\right)}^{2}+{\left(y_{1}-2{x^{\prime }}_{1}\right)}^{2}}}{1-x_{1}^{2}+y_{1}^{2}}\cdot [3x_{1}V-\text{sin}\;\gamma G-2\;\text{cos}\;\gamma S]\\ F_{y}=\sigma f_{y}=-\sigma \frac{\sqrt{{\left(x_{1}-2{y^{\prime }}_{1}\right)}^{2}+{\left(y_{1}-2{x^{\prime }}_{1}\right)}^{2}}}{1-x_{1}^{2}+y_{1}^{2}}\cdot [3y_{1}V+\text{cos}\;\gamma G-2\;\text{sin}\;\gamma S] \end{array}\right. \end{array}$$ (13)

where *σ* is Sommerfeld number, which is denoted as *σ* = *μωRL*(*R*/*C*)^2^(*L*/2*R*)^2^. *μ* means the viscosity of the oil in sliding bearing, *R* and *C* are the radius of the journal and the clearance between the shaft and bearing, respectively. *L* is the bearing length, *f*_*x*_ and *f*_*y*_ are both the dimensionless form of nonlinear oil film force. *γ*, *V*, *G* and *S* are calculated as

$$\left\{ \begin{array}{c} \gamma ={\text{tan}}^{-1}\frac{y_{1}+2{x^{\prime }}_{1}}{x_{1}-2{y^{\prime }}_{1}}-\frac{\pi }{2}\text{sin}\frac{y_{1}+2{x^{\prime }}_{1}}{x_{1}-2{y^{\prime }}_{1}}-\frac{\pi }{2}\text{sin}\left(y_{1}+2x^{\prime }\right)\\ V=\frac{2}{1-x_{1}^{2}-y_{1}^{2}}+\frac{y_{1}\;\text{cos}\;\gamma -x_{1}\;\text{sin}\;\gamma }{1-x_{1}^{2}-y_{1}^{2}}\\ G=\frac{2}{\sqrt{1-x_{1}^{2}-y_{1}^{2}}}\left(\frac{\pi }{2}+{\text{tan}}^{-1}\frac{y_{1}\;\text{cos}\;\gamma -x_{1}\;\text{sin}\;\gamma }{\sqrt{1-x_{1}^{2}-y_{1}^{2}}}\right)\\ S=\frac{x_{1}\;\text{cos}\;\gamma +y_{1}\;\text{sin}\;\gamma }{1-{\left(x_{1}\;\text{cos}\;\gamma +y_{1}\;\text{sin}\;\gamma \right)}^{2}} \end{array}\right.$$

### Cracked rotor

For the slant crack rotor, although the crack breathing process under torsional vibration excitation is dominated by the excitation frequency *ω*_T_, while the lateral stiffness of the slant crack remains progressively varying with the rotational frequency when there is no torsional excitation [10]. Moreover, the simple cosine model is completely capable of describing the transverse stiffness of slant cracks [7, 10, 13]. Therefore, the cosine model is used to describe the stiffness variation in research, where the rotation angle is given by *β* = tan(*y*_2_/*x*_2_).

**Fig 2:**
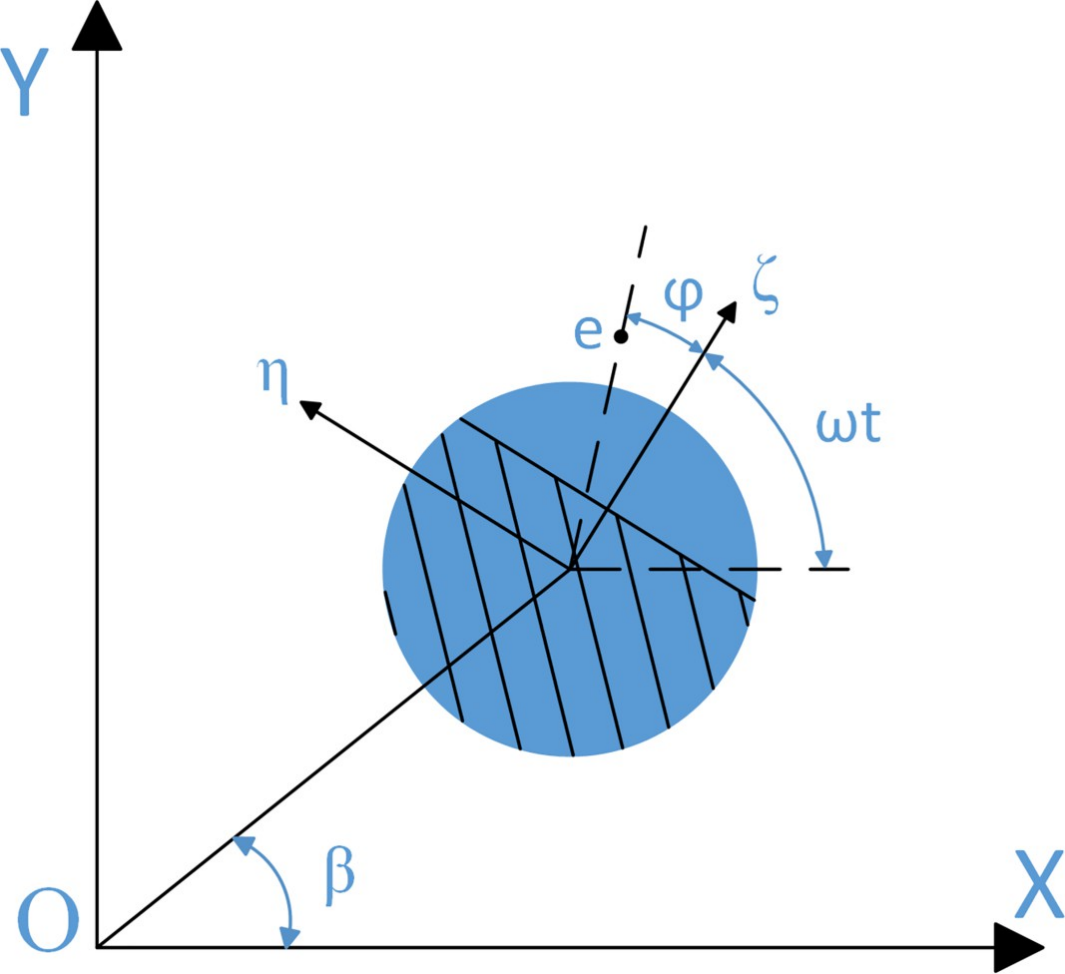


The rotation angle *β* of the shaft is given in a schematic view of the cracked shaft, which is shown in Fig 2. Where *X*_2_*O*_2_*Y*_2_ and *ζO*_1_*η* are the fixed coordinate system and the rotating coordinate system. *ζ* is the direction of the crack.

The stiffness reduction in the *ζ* and *η* directions is Δ*k*_*ζ*_ and Δ*k*_*ζ*_, respectively, if the diagonal crack is assumed to be fully open. Then, *χ* and *α* can be obtained as

$$\begin{array}{c} \chi =\frac{\Delta k_{\zeta }-\Delta k_{\eta }}{\Delta k_{\zeta }+\Delta k_{\eta }} \end{array}$$ (14)

$$\begin{array}{c} \alpha =\frac{\Delta k_{\zeta }+\Delta k_{\eta }}{2k_{0}} \end{array}$$ (15)

where *χ* is the anisotropy ratio of the rotor, *α* = *a*/*D* is the crack depth ratio, and the Eqs (14) and (15) are solved jointly to obtain

$$\begin{array}{c} \left\{ \begin{array}{c} \Delta k_{\zeta }=\alpha k_{0}+\alpha \chi k_{0}\\ \Delta k_{\eta }=\alpha k_{0}-\alpha \chi k_{0} \end{array}\right. \end{array}$$ (16)

The stiffness matrix of the cracked shaft in the rotated coordinate system at this point is shown as

$$\begin{array}{c} \left[K^{\prime }\right]=[\begin{array}{cc} k_{0} & 0\\ 0 & k_{0} \end{array}]-h[\begin{array}{cc} \Delta k_{\zeta } & 0\\ 0 & \Delta k_{\eta } \end{array}] \end{array}$$ (17)

Converting the stiffness matrix of the cracked axis to a fixed coordinate system yields as follows

$$\begin{array}{c} \left[K\right]=[\begin{array}{cc} k_{0} & 0\\ 0 & k_{0} \end{array}]-h[\begin{array}{cc} \Delta k_{\zeta }{\text{cos}}^{2}\left(\omega t\right)+\Delta k_{\eta }{\text{sin}}^{2}\left(\omega t\right) & \left(\Delta k_{\zeta }-\Delta k_{\eta }\right)\text{sin}\left(\omega t\right)\text{cos}\left(\omega t\right)\\ \left(\Delta k_{\zeta }-\Delta k_{\eta }\right)\text{sin}\left(\omega t\right)\text{cos}\left(\omega t\right) & \Delta k_{\zeta }{\text{sin}}^{2}\left(\omega t\right)+\Delta k_{\eta }{\text{cos}}^{2}\left(\omega t\right) \end{array}] \end{array}$$ (18)

The non-dimensional dynamic equations of the bearing are given by

$$\begin{array}{c} \tau =\omega t,X_{1}=\frac{x_{1}}{C},{\dot{X}}_{1}=\frac{{\dot{x}}_{1}}{\omega C},{\ddot{X}}_{1}=\frac{{\ddot{x}}_{1}}{\omega^{2}C},Y_{1}=\frac{y_{1}}{C},{\dot{Y}}_{1}=\frac{{\dot{y}}_{1}}{\omega C},{\ddot{Y}}_{1}=\frac{{\ddot{y}}_{1}}{\omega^{2}C} \end{array}$$

Furthermore, the non-dimensional dynamic equations of the disc are given by

$$\begin{array}{c} \tau =\omega t,X_{s}=\frac{m_{2}g}{k_{0}},\xi =\frac{c}{2\sqrt{k_{0}m_{2}}},\omega_{n}=\sqrt{k_{0}/m_{2}},\Omega =\frac{\omega }{\omega_{n}},e_{1}=\frac{e}{X_{s}},h=\frac{1+\text{cos}(t-\beta +\varphi )}{2},\\ X_{2}=\frac{x_{2}}{X_{s}},{\dot{X}}_{2}=\frac{{\dot{x}}_{2}}{\omega X_{s}},{\ddot{X}}_{2}=\frac{{\ddot{x}}_{2}}{\omega^{2}X_{s}},Y_{2}=\frac{y_{2}}{X_{s}},{\dot{Y}}_{2}=\frac{{\dot{y}}_{2}}{\omega X_{s}},{\ddot{Y}}_{2}=\frac{{\ddot{y}}_{2}}{\omega^{2}X_{s}},\lambda =\frac{X_{s}}{C}=\frac{m_{2}g}{k_{0}C} \end{array}$$

Substituting Eqs (13), (16) and (18) into Eq (1) yields the dimensionless equations for the rotor-bearing system, which are calculated as

$$\begin{array}{c} \left\{ \begin{array}{c} {\ddot{X}}_{1}+\frac{c_{1}{\dot{X}}_{1}}{m_{1}\omega }+\frac{k_{0}}{2m_{1}\omega^{2}}\left(X_{1}-\lambda X_{2}\right)=\frac{F_{x}}{m_{1}C\omega^{2}}\\ {\ddot{Y}}_{1}+\frac{c_{1}{\dot{Y}}_{1}}{m_{1}\omega }+\frac{k_{0}}{2m_{1}\omega^{2}}\left(Y_{1}-\lambda Y_{2}\right)=\frac{F_{y}}{m_{1}C\omega^{2}}-\frac{g}{C\omega^{2}}\\ {\ddot{X}}_{2}+\frac{2\xi }{\Omega }{\dot{X}}_{2}+\frac{1}{\Omega^{2}}\left[\alpha \chi h\;\text{cos}\left(2\tau \right)+1-\alpha h\right]\left(X_{2}-X_{1}/\lambda \right)\\ +\frac{1}{\Omega^{2}}\alpha \chi h\;\text{sin}\left(2\tau \right)\left(Y_{2}-Y_{1}/\lambda \right)=e_{1}\;\text{cos}\left(\tau +\varphi \right)\\ {\ddot{Y}}_{2}+\frac{2\xi }{\Omega }{\dot{Y}}_{2}+\frac{1}{\Omega^{2}}\left[1-\alpha \chi h\;\text{cos}\left(2\tau \right)-\alpha h\right]\left(Y_{2}-Y_{1}/\lambda \right)\\ +\frac{1}{\Omega^{2}}\alpha \chi h\;\text{sin}\left(2\tau \right)\left(X_{2}-X_{1}/\lambda \right)=e_{1}\;\text{sin}\left(\tau +\varphi \right)-\frac{1}{\Omega^{2}} \end{array}\right. \end{array}$$ (19)

## Experiment setup

**Fig 3:**
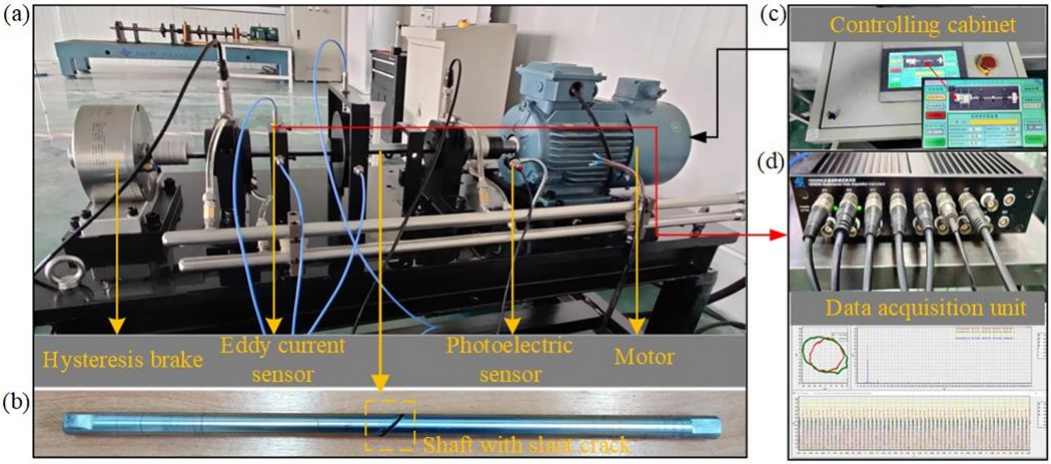


To study the nonlinear response of a rotor characterized by slant cracks subjected to nonlinear oil film forces, a rotor-bearing test bench is constructed, as shown in Fig 3(a), and which undertakes the corresponding experiments. The test bench consists of a hysteresis brake, a eddy current sensor, a photoelectric sensor, a motor and a rotor-bearing system. The total length of bench is 1400 mm. The motor provides the energy source for rotating the rotor with a speed range of 0 to 3500 r/min, while the hysteresis brake carries out the stabilization of linear torque and halts the rotor bearing system. The eddy current sensor and photoelectric sensor allow real-time monitoring of lateral vibrations in both vertical and horizontal directions. The eddy current sensor is connected to a data acquisition unit, which enables the data reading. A 550 mm long shaft is mounted on the test bench and a single span rotor with a diameter of 140 mm, a thickness of 25 mm and a mass of 2.9 kg is attached to the center of shaft. Additionally, the rotor is equipped with threaded holes for the addition of unbalanced masses, making the test bench highly functional.

Based on the constructed rotor-bearing test bench, a diagonal crack was machined in the middle part of the shaft using the cutting machine, as shown in Fig 3(b), to simulate the studied crack type. The diameter of the failed shaft is 20 mm and the crack depth is equal to the shaft radius. Notably, it is necessary to ensure that the direction of the slant crack is located in the key phase position during the installation of the cracked shaft.

The output speed of the motor is regulated by a control cabinet (Fig 3(c)), and the output shaft of the motor is connected to the rotor shaft using a coupling. The motor control shaft rotates at 300 r/min intervals, while the eddy current sensor is set up to monitor the rotor oscillations in the axial and vertical directions, and to collect the nonlinear response of the rotor-bearing system at each constant rotational speed.

Specifically, two eddy current sensors, model WT0150, measure signals from the rotor shaft (near the motor side) and the rotor, respectively. Which are arranged in the axial and vertical directions to feedback the vibration signals in the *X* and *Y* directions, and moreover, the vibration signals in both directions can be coupled and further analyzed. A horizontally placed photoelectric sensor at the coupling is used to react to the rotational speed and phase signals of the rotating shaft. The rotational speed range of the system is 0–3500 r/min, and the valid experimental data can be obtained for 30 s at each rotational speed, with a sampling frequency of 1024 Hz. Subsequently, the signals of vibration displacement at different rotational speeds are acquired by the HD2000 data acquisition system (Fig 3(d)).

## Model validation and result analysis

### Parameter confirmation of rotor-bearing system

The cracked shaft stiffness variation is introduced into the mathematical model of the rotor system, and the fourth-order Runge-Kutta method is employed to solve the differential equations of motion (Eq (19)) of the cracked rotor, to analyze the response of the cracked rotor, to determine the fault characteristics of the cracked shaft, and finally to provide some diagnostic basis for the cracked rotor system. The parameters of the rotor-bearing system are shown in Table 1.

**Table 1:** Parameters of the cracked rotor bearing system.

Quantity	Symbol	Value
Bearing weight	*m*_1_	0.6 kg
Rotor weight	*m*_2_	2.9 kg
Stiffness	*k*_0_	1.2×10^6^ N/m
Bearing length	*L*	0.025 m
Bearing damping	*c*_1_	600 kg/s
Rotor damping	*c*_2_	600 kg/s
Bearing radial clearance	*C*	0.15 mm
Anisotropy ratio	*χ*	0.6
Crack depth ratio	*α*	0.5
Rotor eccentricity	*e*	0.11 mm

Muszynska [24] mentions that, when the 2X harmonics caused by the crack are larger than the 1X harmonics affected by the eccentricity, an inner loop will appear in the cyclonic orbit. However, in the research experiments, the 1X frequency is always dominant, yet a small inner loop appears in the whirl track. Therefore, a larger rotor eccentricity is utilized in the simulation to verify this phenomenon.

### Model results

Under the influence of nonlinear oil film force, the rotor-bearing system with slant crack produces a corresponding nonlinear response during operation, and to explore this pattern, various research methods, such as bifurcation diagram, spectrum diagram, time history, and whirl orbits, have been applied to analyze and compare the model and experimental data.

**Fig 4:**
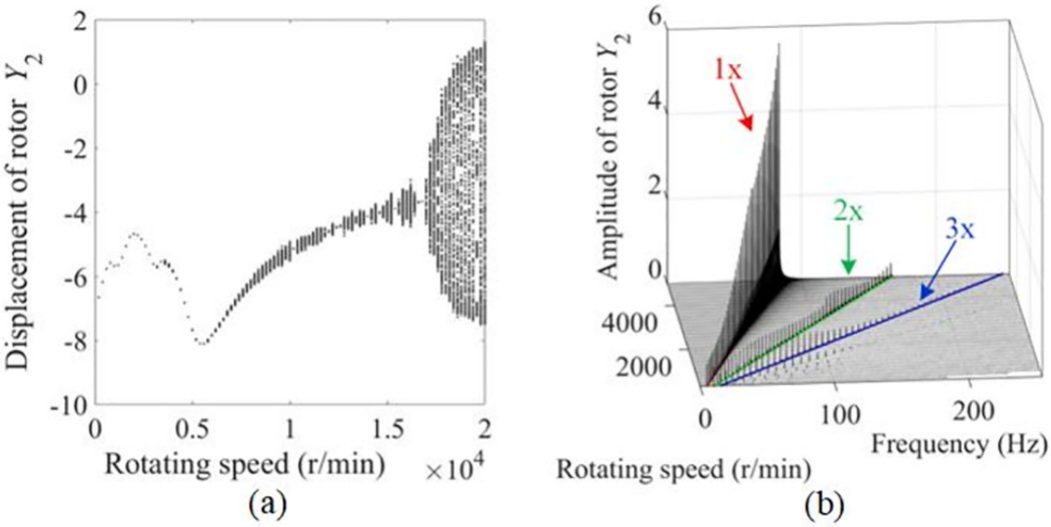


Fig 4(a) shows the bifurcation diagram of the rotor with slant crack rotor obtained from the mathematical model of the rotor-bearing system. As shown in Fig 4(a), the system is mostly in period-1 when the rotational speed is lower than the critical speed of 6143 r/min. The bifurcation phenomenon occurs only when the system’s rotational speed is in the range of 3400 r/min to 3800 r/min, and system then returns to period-1 again after a short period of multi-period motion. However, when the system rotational speed exceeds the critical speed of the system, the rotor system’s motion appears to bifurcate obviously and continuously, and gradually enters into chaos. Moreover, multiple peaks in the point set can be observed from Fig 4(a), and the peaks are most prominent when the system rotational speed is close to 2100 r/min (1/3 of the critical rotational speed), which indicate that the rotor system’s operating state has changed greatly at this time and resonance phenomenon has occurred.

Fig 4(b) shows the waterfall diagram of the cracked rotor at various rotational speeds, where the positions of 1x, 2x and 3x frequencies are labeled in red, green and blue, respectively. As can be seen in Fig 4(b), the 2x frequency component of the rotor system is evident at subcritical speeds, which is caused by the anisotropy of the rotor with slant cracks. Additionally, the 2x and 3x frequency components are relatively large and a small amount of 4x frequency is captured in the spectrogram near the 1/3 critical speed of the system.

**Fig 5:**
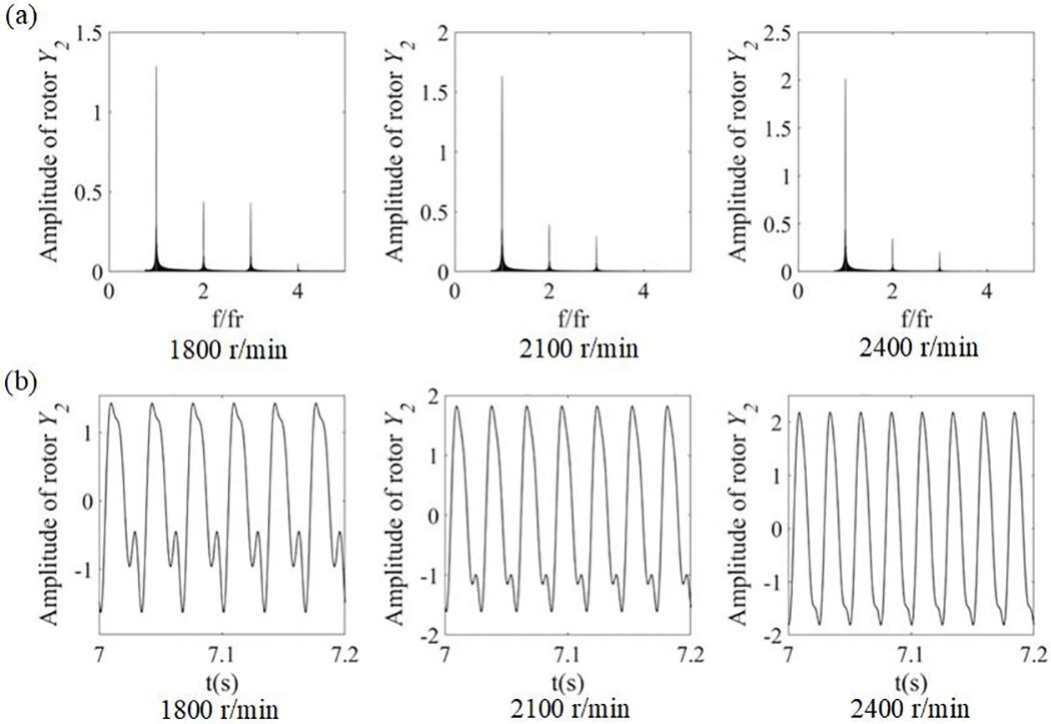


Fig 5 presents the spectrum diagrams and time histories obtained from the mathematical model for speeds of 1800 r/min, 2100 r/min and 2400 r/min. As can be seen from the spectrogram shown in Fig 5(a), 4x frequency components are indeed observed near the 1/3 critical speed, which is consistent with the conclusion drawn from Fig 5(b). And the frequency of the system increases gradually with increasing rotational speed. And from the time histories of the rotor bearing system, it can be seen that the nonlinear response of the system is periodic, but the curve is affected by the crack breathing, which has a depression in the negative direction and produces two troughs in each cycle. Then, the curve becomes smoother as the speed increases.

**Fig 6:**
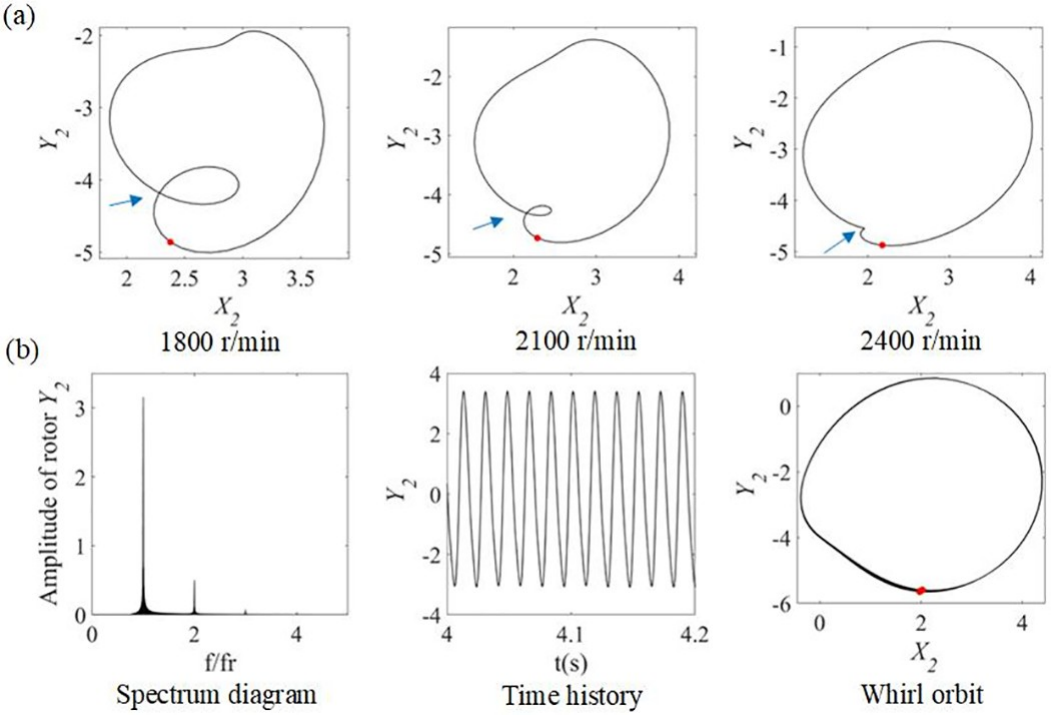


Whirl orbits of the system at speeds of 1800 r/min, 2100 r/min and 2400 r/min are further plotted, as shown in Fig 6(a). It is clearly observed that near 1/3 of the critical speed, the Whirl orbits appear as inner loop, depression occurs in other phases and stretching occurs horizontally. As the speed increases in the range of 1800 r/min to 2400 r/min, the depression gradually eases, the inner ring narrows down and its position rotates counterclockwise by a small angle.

To analyze the nonlinear response of the rotor-bearing system comprehensively, the beyond-critical speed of 3400 r/min is studied and the spectrum diagram, time history and whirl orbit are plotted at this speed, which are shown in Fig 6(b). When the motion of the rotor system bifurcates, the 2x frequency component still occupies a certain proportion, yet the time shows a strong periodicity and the defects of the curve disappear completely. In the whirl orbit of Fig 6(b), the Ponchelet mapping of the rotor is marked with red dots. The point spacing of the Ponchelet mapping and the slight stretching of the whirl orbit curves indicate that, the rotor system motion is more stable and does not have reliable fault characteristics. Therefore, the maximum speed of the experiment is limited to 2500 r/min for safety reasons.

### Experimental results and comparison with model results

**Fig 7:**
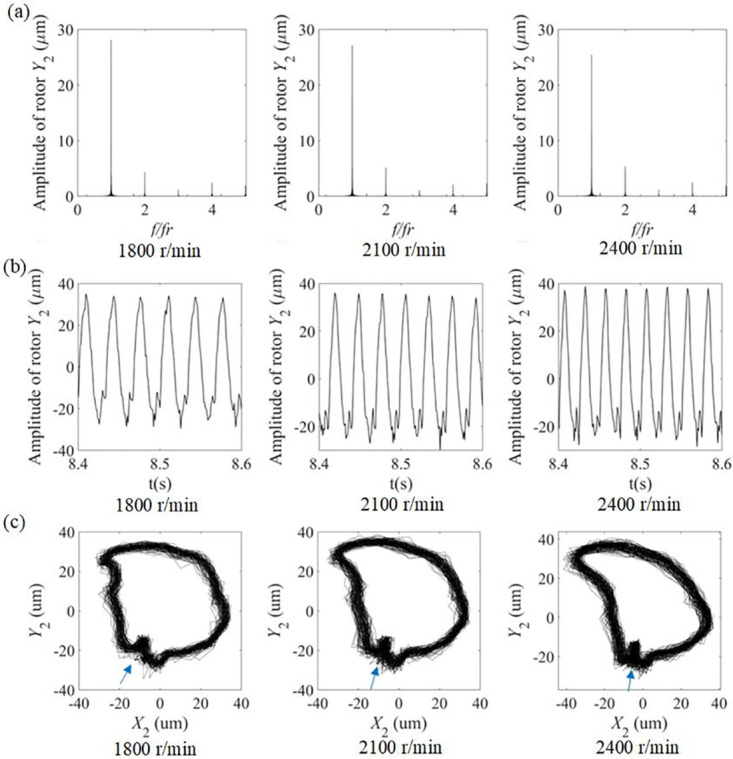


Fig 7(a) shows the spectrum of the rotor near 1/3 critical speed in the experiment, it can be found that the vibration signal of the rotor has 2x, 3x, 4x and other frequency components apart from the working frequency. The 2x frequency components are larger compared to other higher harmonic frequencies, which fully illustrates the asymmetry of the cracked rotor stiffness.

The experimental time histories for the rotor are also shown in Fig 7(b), it can be noticed that the motion of the rotor-bearing system has a certain periodicity and the system is relatively stable. Which is the same as the calculation result of the model, the curve also has a depression in the negative direction and there are two valleys in the negative direction. This is basically consistent with the phenomenon in the numerical model.

Fig 7(c) shows the experimental whirl orbits of the rotor. It can be observed that the whirl orbit has an obvious concave and convex phenomenon and there is always an inner loop. Compared with the model results, the position of the inner loop has a small change similar to that. Though no obvious change in the size of the inner loop can be observed, the curve near the inner loop appears to ‘contract’ when the speed increases.

**Fig 8:**
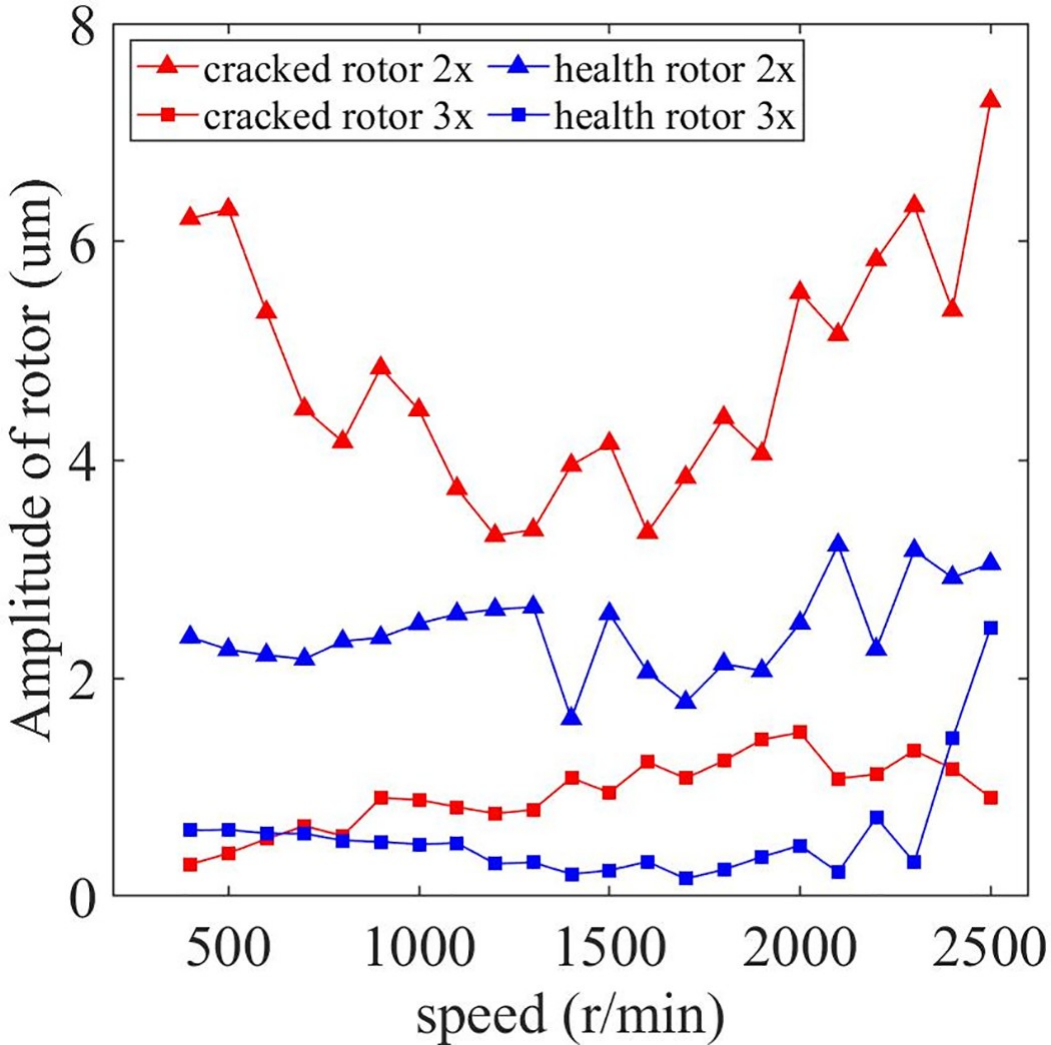


The variation of amplitude with rotational speed is further compared between the cracked and normal (health) rotors, as showm in Fig 8. It can be found that the 2x and 3x frequencies of the cracked rotor have a large enhancement compared to the normal operating conditions. Moreover, the 2x frequency difference between cracked rotor and normal operating conditions is more pronounced compared to the 3x frequency. Thus, the magnitude and change of 2x frequency near 1/3 critical speed of this text bench can be used as an important influencing factor for the preliminary judgment of the cracked rotor motion state.

## Conclusion

In this paper, considering the combined effects of nonlinear oil film forces and cracks on the rotor-bearing system, a study and comparative analysis were conducted using models and experiments, and the results proved that The cracked rotor-bearing system is relatively stable at subcritical speeds. The system only shows a relatively obvious bifurcation in a small range of speeds near 3600 r/min and is in a period-1 at all other speeds. Moreover, for the anisotropy of the stiffness at the crack in the rotor system, there is always a more obvious 2x frequency component in the rotor’s frequency spectrum. And it can be observed in the rotor’s time-history diagram, the negative direction of the rotor time course curve appears depression and makes two valleys in each cycle. Corresponding to the rotor’s frequency and time-history characteristics, an inner loop appears in the whirl orbits near 1/3 of the critical speed. With the increasing speed, the inner loop gradually shrinks and its position rotates counterclockwise by a small angle. In experiments, though no obvious change in the size of the inner loop can be observed, the curve near the inner loop appears to “contract” when the speed increases.

The research results can provide a reference for the life prediction and assessment, the optimization of equipment operation methods, and the improvement of equipment reliability and safety for rotating equipment such as generators, turbines and wind turbines. Moreover, for aerospace equipment such as aircraft engines, turbomachinery, and jet engines, the findings can help improve the performance and safety of the vehicles, reduce the risk of accidents, and guide repair and maintenance strategies.

Moreover, in this study, slant-cracked rotors considering nonlinear oil film forces are investigated. Actually, the rotor-bearing system is also subjected to unbalanced magnetic pull, which is caused by the eccentricity of the mass in the rotor and the inhomogeneity of the air gap between the stator and rotor. For the following researches, the unbalanced magnetic pull can be introduced into the system to restore the actual situation more. Furthermore, the effect of other cracks, such as transverse and longitudinal cracks, on the system can be investigated.

## Supporting information

S1 File Experimental original data after compression. These data were measured by two eddy current sensors, model WT0150.
(ZIP)
